# PI3K inhibition synergizes with glucocorticoids but antagonizes with methotrexate in T-cell acute lymphoblastic leukemia

**DOI:** 10.18632/oncotarget.3524

**Published:** 2015-04-01

**Authors:** André Bortolini Silveira, Angelo Brunelli Albertoni Laranjeira, Gisele Olinto Libanio Rodrigues, Paulo César Leal, Bruno António Cardoso, João Taborda Barata, Rosendo Augusto Yunes, Nilson Ivo Tonin Zanchin, Sílvia Regina Brandalise, José Andrés Yunes

**Affiliations:** ^1^ Laboratório de Biologia Molecular, Centro Infantil Boldrini, Campinas, SP, Brazil; ^2^ Departamento de Química, Universidade Federal de Santa Catarina, Florianópolis, SC, Brazil; ^3^ Instituto de Medicina Molecular, Faculdade de Medicina da Universidade de Lisboa, Lisboa, Portugal; ^4^ Instituto Carlos Chagas, Fundação Oswaldo Cruz, Curitiba, PR, Brazil; ^5^ Departamento de Genética Médica, Faculdade de Ciências Médicas, UNICAMP, Campinas, SP, Brazil

**Keywords:** PI3K, AS605240, T-ALL, drug resistance, glucocorticoids

## Abstract

The PI3K pathway is frequently hyperactivated in primary T-cell acute lymphoblastic leukemia (T-ALL) cells. Activation of the PI3K pathway has been suggested as one mechanism of glucocorticoid resistance in T-ALL, and patients harboring mutations in the PI3K negative regulator *PTEN* may be at increased risk of induction failure and relapse. By gene expression microarray analysis of T-ALL cells treated with the PI3K inhibitor AS605240, we identified Myc as a prominent downstream target of the PI3K pathway. A significant association was found between the AS605240 gene expression signature and that of glucocorticoid resistance and relapse in T-ALL. AS605240 showed anti-leukemic activity and strong synergism with glucocorticoids both *in vitro* and in a NOD/SCID xenograft model of T-ALL. In contrast, PI3K inhibition showed antagonism with methotrexate and daunorubicin, drugs that preferentially target dividing cells. This antagonistic interaction, however, could be circumvented by the use of correct drug scheduling schemes. Our data indicate the potential benefits and difficulties for the incorporation of PI3K inhibitors in T-ALL therapy.

## INTRODUCTION

T-cell acute lymphoblastic leukemia (T-ALL) is an aggressive hematologic cancer [1]. The prognosis of children with T-ALL has improved with modern treatment protocols, based on the use of aggressive multi-agent therapies [2]. However, T-ALL patients displaying chemotherapy resistance show very poor survival. Accumulating evidence indicates that the PI3K pathway is linked to resistance to therapy in several types of cancer [3].

Class I PI3Ks are a family of lipid kinases predominantly activated by protein tyrosine kinases in response to cell surface receptors. PI3K phosphorylates phosphatidylinositol 4, 5-bisphosphate (PIP_2_) generating phosphatidylinositol 3, 4, 5-trisphosphate (PIP_3_). PIP_3_ serves as an anchor for pleckstrin homology (PH) domain-containing proteins, which initiate the assembly of signaling complexes that results in the activation of a plethora of downstream effector molecules, including protein kinase B (Akt) [4] and the mammalian target of rapamycin complex 1 (mTORC1) [5]. The PI3K pathway is central to the control of cell survival, energy metabolism, cellular motility and cell cycle progression [6–7].

Constitutive activation of the PI3K pathway in hematological malignancies has been shown to support tumor cell proliferation, viability and drug resistance *in vitro* [8–10]. Moreover, PI3K signaling pathway is frequently hyperactivated in primary T-ALL cells, for instance due to microenvironmental stimulation, interleukin-7 receptor (*IL7R*) oncogenic mutations or phosphatase and tensin homolog (*PTEN*) inactivation [11–15]. Activation of the PI3K effector mTOR in T-ALL has been suggested to represent one mechanism of resistance to glucocorticoids, a common feature of relapse [16–17]. Moreover, synergistic effects of inhibitors of the PI3K-downstream mTOR pathway in T-ALL cell lines have been described [18–19]. We and others have shown that T-ALL patients harboring *PTEN* mutations may be at increased risk of induction failure and relapse [13, 20]. Importantly, *PTEN* aberrations were associated with poor outcome and relapse in T-ALL [20–22], suggesting that the level of PI3K activation may influence resistance to treatment.

In this study, T-ALL cells were treated with a PI3K inhibitor to identify a transcriptional PI3K activity signature. PI3K inhibition downregulated genes associated with cellular growth and targets of Myc. Moreover, the comparison of the PI3K signature with gene expression data of primary T-ALL samples indicates that higher PI3K activity is associated with glucocorticoid resistance and worse clinical outcome. We opted to use the PI3K inhibitor AS605240 in light of its favorable pharmacological and biochemical characteristics [23–24]. This allowed us to test the long term inhibition effects of PI3K in a NOD/SCID xenograft model of T-ALL. Functional assays demonstrated that PI3K inhibition sensitizes T-ALL cells to glucocorticoids, but antagonizes methotrexate (MTX) and daunorubicin (DNR), unless correct drug scheduling is used.

## RESULTS

### PI3K activity is associated with increased chemotherapy resistance and poor prognosis in T-ALL

Most cell lines are maintained in culture for years and accumulate several genetic lesions not characteristic of primary disease [25]. On the other hand, ALL primary cells do not divide *in vitro* [26], which may affect their response to small molecules [27]. Hence, we decided to obtain transcriptional signatures of PI3K activity from both cell lines and primary cells, which would provide complementary aspects of gene expression modulation by PI3K. To do so, seven T-ALL cell lines [GSE50998] and 15 diagnostic T-ALL patient samples [GSE51000] were treated with the PI3K inhibitor AS605240 or vehicle for 6 h, and subjected to global gene expression analysis using whole-transcript Affymetrix expression arrays. Principal Component Analysis (PCA) showed that most samples responded similarly to PI3K inhibition, irrespectively of *PTEN* and *NOTCH1* mutational status (Supplementary Figure 1). Using paired Limma analysis, we obtained 211 genes downregulated and 78 genes upregulated in T-ALL primary cells (adjusted *p*-value < 0.05, FC > 1.5; Supplementary Table 1) and 174 genes downregulated and 395 genes upregulated in T-ALL cell lines (adjusted *p*-value < 0.05, FC > 1.5; Supplementary Table 2) in response to AS605240 treatment. As expected, Connectivity Map analysis showed that the signature resulting from PI3K inhibition in T-ALL cell lines correlated with signatures of classical PI3K inhibitors (wortmannin and LY-294002), and rapamycin (sirolimus; Supplementary Figure 2). Rapamycin is an inhibitor of mTORC1, a complex downstream of PI3K signaling [5]. Although other interpretations may be possible, genes downregulated by AS605240 were here considered to be under positive transcriptional control of PI3K, whereas those upregulated by AS605240 were considered under negative control of PI3K.

**Figure 1:**
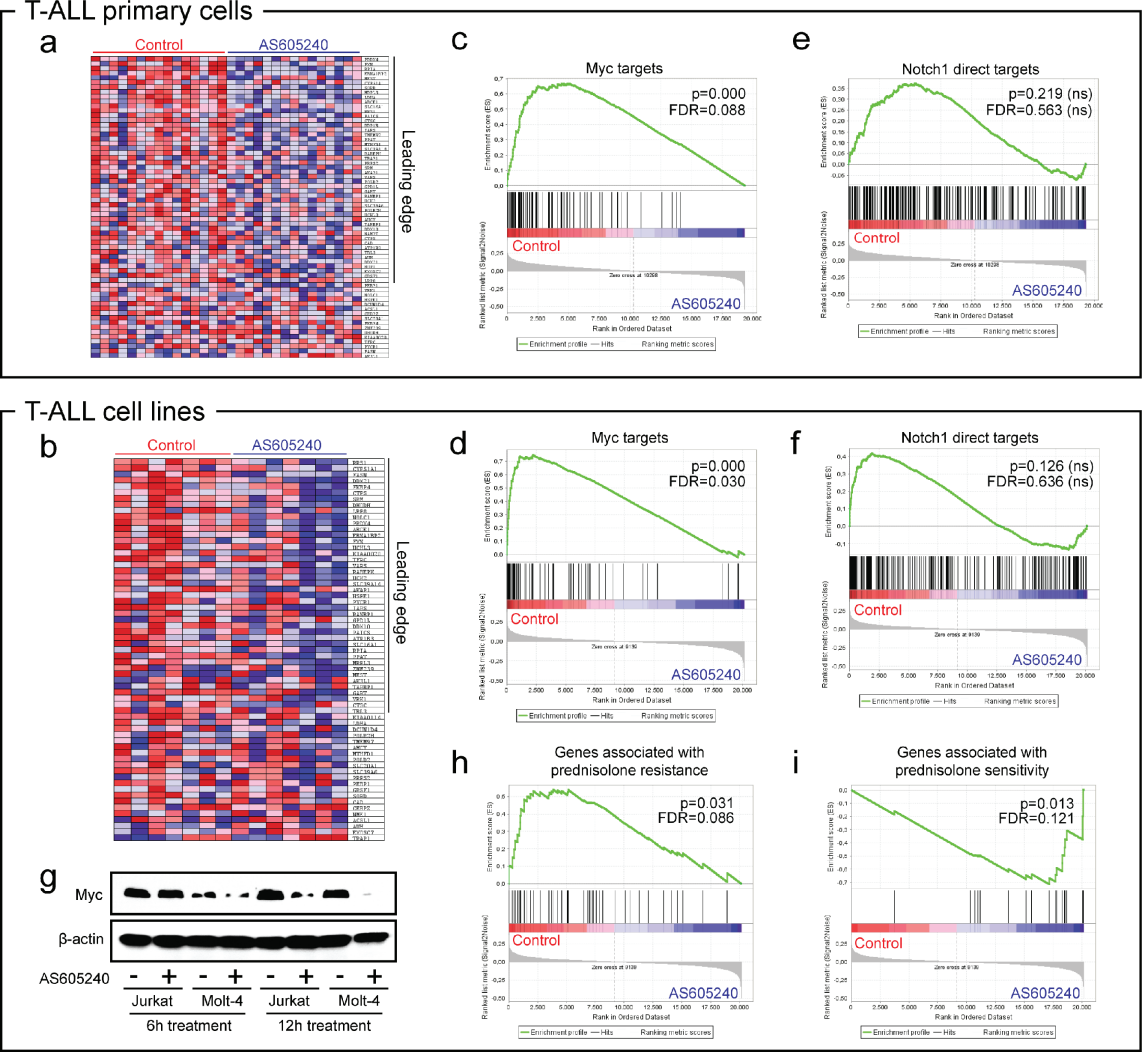
PI3K inhibition downregulates Myc in a Notch1-independent manner and is associated to glucocorticoid sensitivity GSEA heatmaps **(a–b)** and enrichment plots **(c–d)**. Targets of Myc [28–30] are downregulated in T-ALL cell lines (CCRF-CEM, HPB-ALL, Jurkat, Molt-4, P12-ICHIKAWA, ALL-SIL and TALL-1, from left to right in heatmaps) and primary cells upon treatment with the PI3K inhibitor AS605240 for 6 h. We show representative plots corresponding to the gene set described in Schuhmacher et al., 2001 (SCHUHMACHER_MYC_TARGETS_UP) [30]. Additional plots are provided in Supplementary Figure 3. In heatmaps, red and blue tones indicate higher or lower expression, respectively. Leading edge genes are indicated. **(e–f)** GSEA enrichment plots. Direct targets of Notch1 [34] were not significantly modulated by AS605240. **(g)** AS605240 inhibits accumulation of Myc protein. Western blotting of Myc and β-actin of Jurkat and Molt-4 cells treated with the IC_50_ values of AS605240 or vehicle for 6 h and 12 h. **(h–i)** Genes discriminative for prednisolone resistance [39] were downregulated in AS605240-treated cell lines and genes discriminative for prednisolone sensitivity [39] were upregulated in AS605240-treated ones.

GSEA analysis showed that AS605240 treatment downregulated several gene sets related to cellular growth in both T-ALL cell lines and primary cells, including energy metabolism (glucose transport and metabolism, oxidative phosphorylation, pyruvate metabolism, and TCA cycle), transcription (nucleotide metabolism, RNA polymerase III transcription, tRNA and miRNA biosynthesis) and biosynthesis of a plethora of compounds and cellular structures (biosynthesis of purines, pyrimidines, amino acids, glucose, fatty acids and steroids, and biogenesis of lysosome vesicles and mitochondria). Among the top tier of gene sets transcriptionally downregulated by AS605240 in both cell lines and primary cells, four different gene sets represented targets of Myc (Figure 1a–1d; Supplementary Figure 3a–b) [28–30]. A fifth gene set representing a Myc oncogenic signature [31] was downregulated by AS605240 in cell lines (Supplementary Figure 3b).

The transcription factor Myc is a master regulator of cell growth and multiple biosynthetic and metabolic pathways [32], and its expression correlates with leukemia initiating activity in T-ALL [33]. Myc inhibition by AS605240 appeared to be Notch1-independent, since Notch1 direct targets [34] were not transcriptionally modulated (Figure 1e–1f). On the other hand, a broader gene set of Notch1-responsive genes, which includes several Myc targets [35], was significantly downregulated in cell lines treated with AS605240 (Supplementary Figure 3c). Although *MYC* mRNA levels were not significantly altered after 6 h of AS605240 treatment (data not shown), western blotting analysis evidenced decreased Myc protein levels in Jurkat and Molt-4 cells after PI3K inhibition (Figure 1g). Quantitative PCR confirmed downregulation of Myc targets *ALOX5AP*, *BCAT1*, *PRDX4* and *LDHA* in primary cells treated with AS605240 (Supplementary Figure 3d). *BCAT1* was found to induce cell proliferation, migration and invasion in nasopharyngeal carcinoma [36]. *LDHA* and *PRDX4* were described as overexpressed in high-risk neuroblastomas independently of other markers [37–38].

Ingenuity Pathway Analysis showed that the top biological functions downregulated by AS605240 in both cell lines and primary cells were related to cholesterol biosynthesis (Supplementary Figure 4). Glucocorticoid resistance in T-ALL has been associated to the upregulation of genes linked to cellular respiration, biosynthetic and metabolic pathways, proliferation and Myc. Notably, genes responsible for cholesterol biosynthesis were found highly upregulated in prednisolone resistant T-ALL [39], and ALL cells were shown to be particularly dependent on endogenously synthesized cholesterol, which is essential for the synthesis of cellular membranes of highly proliferative cells [40]. Because PI3K inhibition targeted genes involved in Myc signaling, cellular growth, and cholesterol biosynthesis, we hypothesized that the AS605240-derived signature would be correlated with gene expression patterns of glucocorticoid resistance in T-ALL. Indeed, GSEA analysis showed that genes associated with prednisolone resistance [39] were downregulated due to PI3K inhibition (Figure 1h), whereas those associated with prednisolone sensitivity [39] were upregulated (Figure 1i).

**Figure 2:**
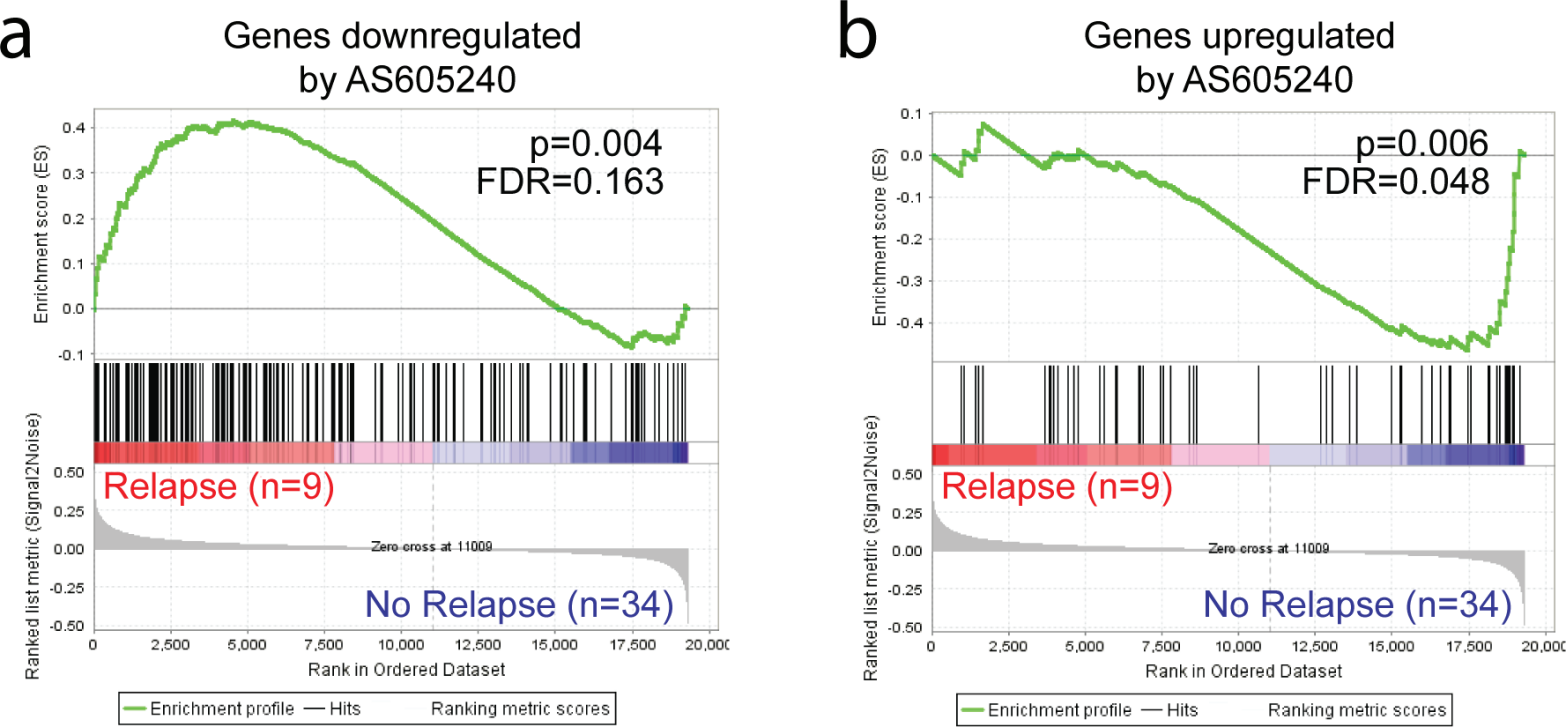
Genes responsive to PI3K inhibition show differential expression in T-ALL samples of patients who underwent relapse Diagnostic T-ALL samples of patients who underwent relapse showed **(a)** higher expression of genes downregulated by AS605240 and, conversely, **(b)** lower expression of genes upregulated by AS605240 in primary cells *in vitro*.

The PI3K activity signatures were then compared against expression microarray data of 43 primary T-ALL samples obtained at diagnosis from patients treated under the GBTLI ALL-99 [41] and GBTLI-2009 protocols at Centro Infantil Boldrini [GSE50999] (for clinical and biological data, refer to Supplementary Table 3). GSEA analysis showed that patients that underwent relapse had higher expression of genes under positive control of PI3K (downregulated by AS605240) and lower expression values of genes under negative control of the pathway (upregulated by AS605240; Figure 2). Altogether, our gene expression data suggest that higher PI3K activity may be associated with increased glucocorticoids resistance and poor prognosis, which is in accordance with previous data showing that *PTEN* mutated patients may be at increased risk of early treatment failure and relapse [13, 20].

### The PI3K inhibitor AS605240 shows anti-leukemic activity and prevents leukemic progression in T-ALL engrafted NOD/SCID mice

**Figure 3:**
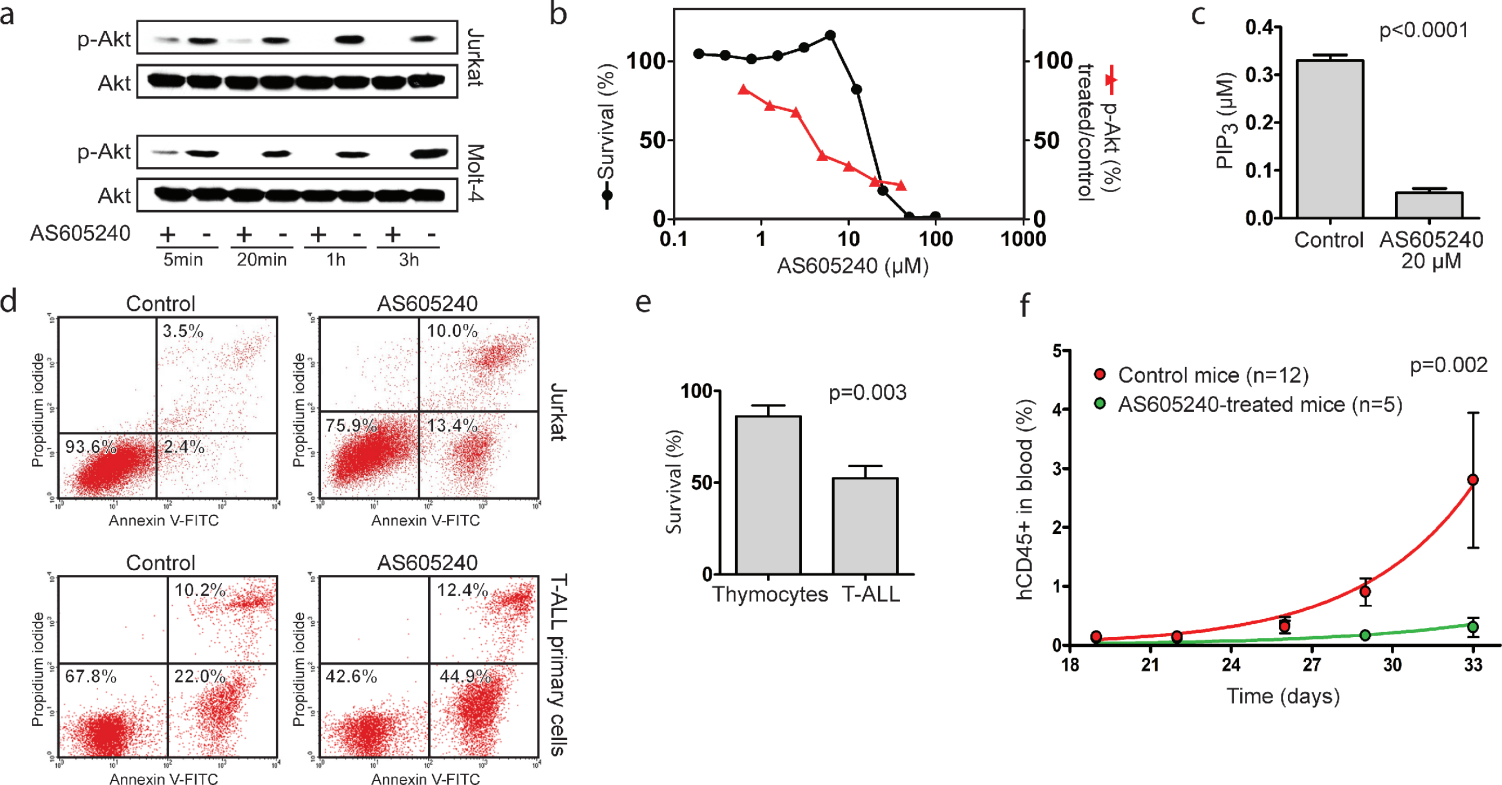
AS605240 inhibited p-Akt and PIP3 accumulation in T-ALL and showed anti-leukemic activity both *in vitro* and *in vivo* **(a)** Western blotting analysis for Akt and p-Akt (Ser473) in Jurkat and Molt-4 cells treated with AS605240 IC_50_ values or vehicle. **(b)** Effect in cell survival and p-Akt accumulation of increasing concentrations of AS605240 in Jurkat cells. Survival was assessed by the MTT assay after 96 h of incubation. Quantification of p-Akt was performed with the AKT [pS473] ELISA Kit (Invitrogen) after 24 h of incubation. **(c)** AS605240 inhibits PIP_3_ accumulation, as assessed by PIP_3_ mass ELISA quantification in Jurkat cells incubated for 24 h with AS605240 or vehicle. *P*-value obtained with a paired *T*-test. **(d)** AS605240 induces apoptosis, as assessed by Annexin V/PI double staining and flow cytometry analysis in Jurkat and primary T-ALL cells treated with AS605240 or vehicle for 8 h and 6 h, respectively. Additional plots in Supplementary Figure 5. **(e)** Primary T-ALL cells (*n* = 7) are more sensitive to AS605240 than normal human thymocytes (*n* = 7). Cells were incubated with 10 μM of AS605240 for 48 h and survival was assessed by the MTT assay. P-value obtained with an unpaired *T*-test. **(f)** AS605240 prevents leukemic progression in NOD/SCID mice engrafted with primary T-ALL cells. Eleven days after the injection of 10^7^ T-ALL cells, mice started to be treated with 20 mg/Kg of AS605240, intraperitoneally twice a day, 5 days a week. Peripheral blood proportion of human CD45+ cells was measured by flow cytometry analysis after red blood cells lysis. Exponential growth curves were compared with the F test of the best-fit K values. Error bars indicate the standard error of the mean (SEM).

Next, we sought to characterize the effects of AS605240 against T-ALL cells, with the goal of pre-clinically assessing the potential benefits of PI3K inhibition in T-ALL. AS605240 inhibited p-Akt (Figure 3a–3b) and PIP_3_ (Figure 3c) accumulation and promoted apoptosis in both T-ALL cell lines and primary cells, with activation of Caspase-3 (Figure 3d, Supplementary Figure 5). The IC_50_ concentrations of AS605240 in a 96 h assay ranged from 12 to 22 μM depending on the cell line tested (Figure 3b, Supplementary Figure 6). These values are within the same range necessary to inhibit approximately 75% of PIP_3_ accumulation in Jurkat cells (Figure 3b). The lower survival plateau reached zero in all cell lines and no correlation was found between IC_50_ concentrations and *PTEN* mutational status (data not shown). Notably, T-ALL primary cells were significantly more sensitive to AS605240 than normal human thymocytes (Figure 3e).

To test the *in vivo* efficacy of AS605240 treatment, NOD/SCID mice were transplanted with primary T-ALL cells derived from one patient sample and randomized eleven days later to receive AS605240 (20mg/kg) or vehicle, intraperitoneally twice a day, 5 days a week. Mice treated with the PI3K inhibitor showed decreased leukemic progression in comparison to control mice, as evaluated by the percentage of human CD45+ cells in peripheral blood at different time points (Figure 3f).

### AS605240 synergizes with glucocorticoids both *in vitro* and in a mouse xenograft model of T-ALL

**Figure 4:**
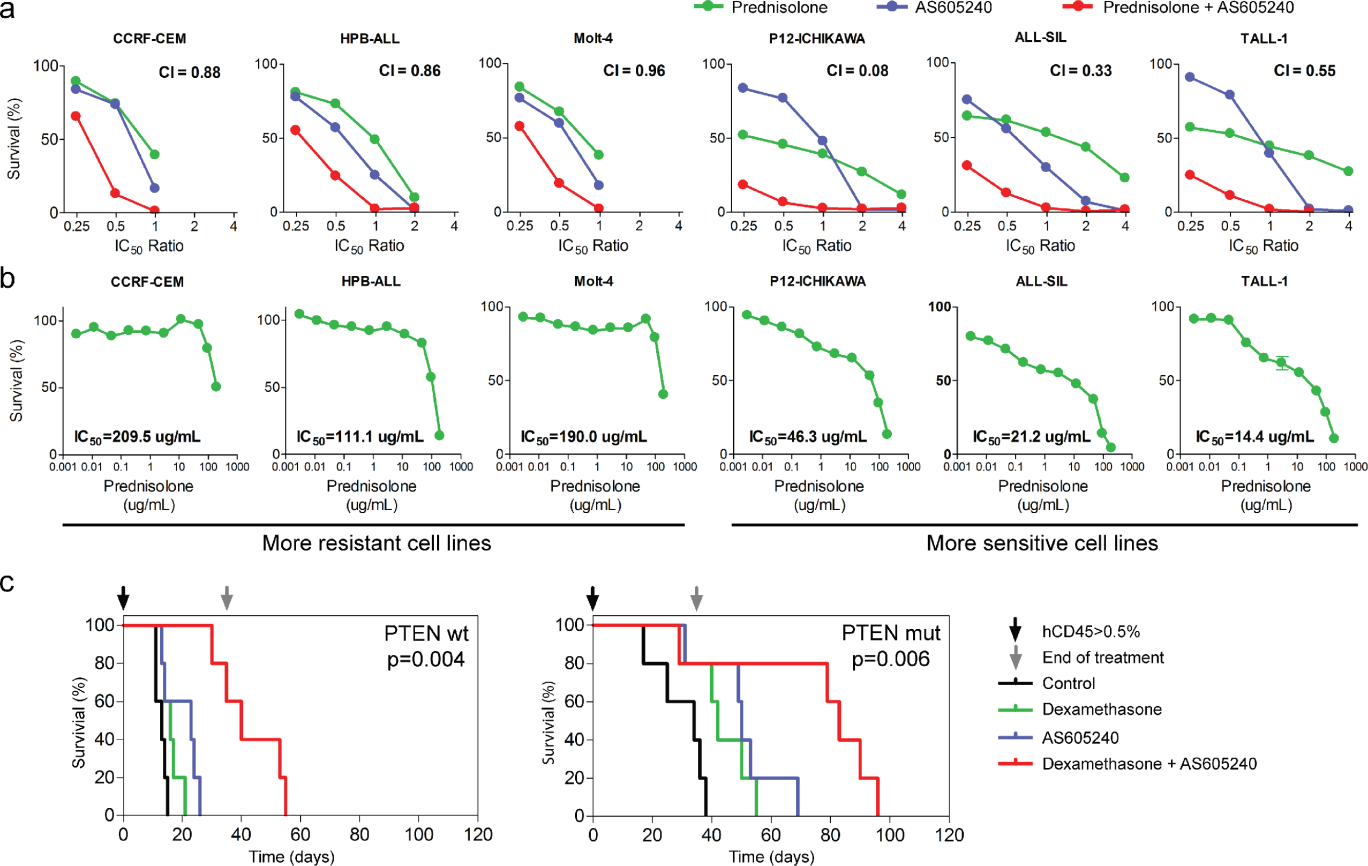
AS605240 shows a synergistic interaction with prednisolone **(a)** MTT assay of T-ALL cell lines treated for 96 h with several ratios of the IC_50_ values of AS605240, prednisolone or both. Combination Index (CI) values are indicated. Synergistic effect is characterized by CI < 0.9 and additive effect by 0.9 > CI > 1.1. **(b)** MTT assay of T-ALL cell lines treated for 96 h with increasing concentrations of prednisolone. **(c)** NOD/SCID mice engrafted with primary T-ALL cells were treated with 30 mg/Kg of AS605240 and/or 5 mg/Kg of dexamethasone, intraperitoneally once a day, 5 days a week. Treatment started when human CD45+ accounted for more than 0.5% of peripheral blood cells after red blood cells lysis.

To further evaluate the clinical potential of PI3K inhibition for T-ALL treatment, we measured the cytotoxic effect of AS605240 in combination with commonly used chemotherapeutic drugs (Table 1). We observed a synergistic interaction between AS605240 and a glucocorticoid (prednisolone) in 5 out of 6 cell lines tested (Figure 4a). Notably, cell lines intrinsically more resistant to prednisolone (CCRF-CEM, HPB-ALL and Molt-4) showed weaker synergism between AS605240 and prednisolone, characterized by higher Combination Index (CI) values (Figure 4a–4b). According to the COSMIC database, cell lines CCRF-CEM, Jurkat and Molt-4 show glucocorticoid receptor (*NR3C1*) missense mutations. P12-ICHIKAWA and TALL-1 don't show alterations in *NR3C1.* The mutations identified in CCRF-CEM and Jurkat have been associated with functionally defective glucocorticoid receptors [42–43]. In agreement with that, these cell lines were more resistant to prednisolone than the remaining cell lines analyzed. Jurkat cells showed the highest calculated IC_50_ value of prednisolone. For this cell line, we were unable to measure the CI between prednisolone and AS605240.

**Table 1:** Combination Index (CI) between AS605240 and methotrexate (MTX), prednisolone (Pred), asparaginase (Asp) or daunorubicin (DNR) in T-ALL cell lines MTT assay of cell lines treated for 96h with several ratios of the IC_50_ values of each drug. Combination index (CI) was calculated with the Calcusyn software. Synergistic effect is characterized by CI<0.9, additive effect by 0.9<CI<1.1 and antagonistic effect by CI>1.1. The columns named Pred, Asp, MTX and DNR represent results obtained with the simultaneous use of AS605240 and the respective drug. Columns MTX sched and DNR sched represent the scheduling scheme where AS605240 was added to culture media 48h after the respective drug.

	Combination index (CI)					
Cell line	Pred	Asp	MTX	MTX sched	DNR	DNR sched
**CCRF-CEM**	0.88	0.84	2.91	1.11	1.76	1.67
**HPB-ALL**	0.86	1.48	1.23	1.00	1.07	-
**Jurkat**	-	1.07	-	1.62	2.10	-
**Molt-4**	0.96	1.07	2.55	1.21	1.48	-
**P12-ICHIKAWA**	0.08	0.93	1.78	0.75	1.27	0.89
**ALL-SIL**	0.33	1.35	1.39	0.96	1.12	-
**TALL-1**	0.55	0.94	1.57	1.10	1.63	0.89

Accordingly, NOD/SCID mice engrafted with primary T-ALL cells derived from two patient samples showed enhanced survival when treated with a combination of AS605240 (30 mg/kg once a day) and another glucocorticoid (dexamethasone), in comparison to treatment with either drug alone (Figure 4c). Noteworthy, no difference was observed in weight loss of T-ALL transplanted NOD/SCID mice treated with AS605240 versus dexamethasone (Supplementary Figure 7). In conclusion, our data strongly suggest that PI3K inhibition potentiates the activity of glucocorticoids against T-ALL cells both *in vitro* and *in vivo*.

### PI3K inhibition requires scheduling when combined with drugs that target dividing cells

**Figure 5:**
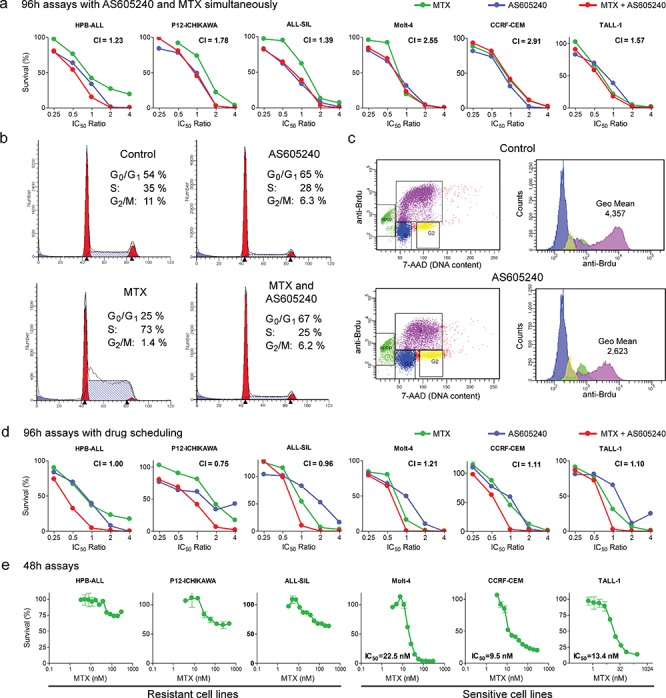
AS605240 showed an antagonistic interaction with MTX, which could be successfully circumvented by means of proper drug scheduling **(a)** AS605240 shows an antagonistic interaction with MTX when drugs are administered concomitantly. MTT assay of T-ALL cell lines treated for 96 h with several ratios of the IC_50_ values of AS605240, MTX or both. Combination index (CI) values are indicated. Synergistic interaction, CI < 0.9; additive interaction, 0.9 < CI < 1.1; antagonistic interaction, CI > 1.1. **(b)** AS605240 reduces the percentage of cells in S and completely abrogates MTX-induced S-phase arrest. Molt-4 cells were incubated with the IC_50_ values of AS605240 and/or MTX for 24 h, propidium iodide stained and analyzed by flow cytometry. **(c)** AS605240 reduces BrdU nucleotide incorporation, as shown by reduced anti-BrdU fluorescence intensity of S-phase cells (pink) in Jurkat, P12-ICHIKAWA and Molt-4 cells. Cell lines were treated with AS605240 IC_50_ value for 24 h. Representative plots of P12-ICHIKAWA cells are shown. **(d)** AS605240 shows an additive/synergistic interaction with MTX when the PI3K inhibitor is added 48 h after those drugs (4 out of 7 cell lines tested) in a 96 h MTT assay. **(e)** MTT assay of T-ALL cell lines treated for 48 h with increasing concentration of MTX. IC_50_ values were calculated with the GraphPad Prism 5 software.

AS605240 showed an antagonistic interaction *in vitro* with both MTX (Table 1; Figure 5a) and DNR (Table 1) in all cell lines tested. MTX is an antimetabolite that allosterically inhibits dihydrofolate reductase (*DHFR*), which participates in the synthesis of tetrahydrofolate, an essential molecule for the biosynthesis of purines, thymidylate and several amino acids [44]. MTX acts specifically during DNA and RNA synthesis, and thus it is cytotoxic during the S-phase of the cell cycle. AS605240 treatment completely abrogated MTX-induced S-phase arrest *in vitro* (Figure 5b). Part of this antagonistic interaction may result from AS605240-induced G_0_/G_1_ cell cycle arrest, which decreases the number of S-phase MTX-susceptible cells (Table 2, Figure 5b). BrdU incorporation assays also showed decreased rate of nucleotide incorporation during S-phase in Jurkat, Molt-4 and P12-ICHIKAWA cells after 24 h treatment with AS605240 (Table 2, Figure 5c). The lower rate of DNA duplication in AS605240-treated cells may induce increased tolerance to lower nucleotide availability under MTX treatment [45]. Altogether, these results indicate that both AS605240-induced cell cycle arrest at G_0_/G_1_ and the lower rate of DNA replication may contribute to the antagonistic interaction between AS605240 and MTX.

**Table 2:** AS605240 induces G_0_/G_1_ cell cycle arrest and reduces BrdU nucleotide incorporation in T-ALL cell lines Each cell line was treated with the IC_50_ value of AS605240 for 24 h and incubated with BrdU for 30 min. Percentage of cells in each phase of the cell cycle and BrdU incorporation were measured with the BD BrdU Flow Citometry Assay Kit and flow cytometry analysis

Cell line	Treatment	G_0_/G_1_ (%)	S (%)	G_2_/M (%)	BrdU in S (Geo Mean)
**Jurkat**	Control	48.1	37.8	7.4	2.736
	AS605240	55.6	3.3	20.7	1.732
**Molt-4**	Control	47.0	45.1	4.1	4.751
	AS605240	63.8	27.1	4.2	3.587
**P12-ICHIKAWA**	Control	45.8	37.5	6.6	4.357
	AS605240	48.3	26.2	9.6	2.623

Based on these evidences, we explored alternative administration schedules to find possible therapeutic windows for AS605240 use in the context of modern ALL treatment protocols, which include MTX and other anticancer drugs that target dividing cells. When AS605240 was added 48 h after MTX and DNR, we observed lower Combination Index values for all cell lines tested. For MTX, a synergistic interaction was present in P12-ICHIKAWA cells and additive interactions were present in 3 out of 7 cell lines tested. For DNR, combination indexes characterizing slight synergism were present in 2 out of 3 cell lines (Table 1, Figure 5d). Noteworthy, when using this schedule, the most synergistic interactions were observed for the cell lines (HPB-ALL, CI = 1.0; P12-ICHIKAWA, CI = 0.76; ALL-SIL, CI = 0.96) found to be resistant to MTX in a 48 h assay (Figure 5e). Overall, our data indicate that scheduling is of major importance when considering the introduction of PI3K inhibitors in T-ALL treatment schemes with chemotherapeutic drugs that require cell division.

## DISCUSSION

Aberrant activation of PI3K signaling pathways has been implicated in many types of cancer [46]. *PTEN* mutations and elevated Akt activity were found to be associated with poor prognosis in solid tumors [47–48] and T-ALL [13, 20]. In this study, we show data indicative of higher PI3K pathway activity in patients who underwent relapse.

Given that PI3Kγ is predominantly expressed in the hematopoietic system [49], the use of a PI3Kγ specific inhibitor could offer an innovative rationale-based therapeutic strategy for T-ALL. AS605240 was previously described as an isoform-selective ATP-competitive inhibitor of PI3Kγ with 30-fold selectivity over PI3K δ and β, and 7.5-fold selectivity over PI3Kα [23]. However, we found that AS605240 did not have cytotoxic effects against T-ALL cells at concentrations expected to inhibit solely PI3Kγ (0.1–0.2 μM) [23], being cytotoxic only at concentrations high enough to inhibit more than 70% of p-Akt accumulation (Figure 3b), presumably disrupting the activity of several PI3K isoforms. All PI3K p110 subunits are highly expressed in primary T-ALL cells (Supplementary Figure 8) [50] and evidence points to roles of PI3Kα, γ and δ in T-ALL leukemogenesis [13, 50], suggesting that multiple isoforms of PI3K might play a role in T-ALL.

Although the concentrations of AS605240 needed to inhibit the PI3K pathway and induce cell death *in vitro* were in the micromolar range, the drug was more selective to leukemic cells than to normal thymocytes. More importantly, the PI3K inhibitor prevented primary T-ALL progression *in vivo* and may be given to mice for months without inducing generalized toxicity [23–24]. Although this indicates the feasibility of AS605240 use to treat T-ALL patients, we observed that mice had mobility difficulties and remained immobile for several minutes after AS605240 administration (Supplementary Video). This raises the possibility of undesirable neurological side effects not previously reported.

This is the first time that gene products downstream of PI3K have been identified in T-ALL. Interestingly, we observed a prominent effect of PI3K inhibition on the expression of Myc target genes. The PI3K/Akt/mTORC1 pathway is known to positively regulate Myc protein abundance. Previous work in T-ALL demonstrated that Akt phosphorylates and deactivates Gsk3-beta, preventing phosphorylation of Myc and its degradation through the ubiquitin/proteasome pathway [51]. mTORC1 phosphorylates S6K1 which in turn enhances translation of Myc mRNA by modulating the phosphorylation of eukaryotic initiation factor eIF4B [52]. At the transcriptional level, in T-ALL, Myc is under control of Notch1 [53]. Our microarray data showed no regulation of Notch1 direct targets in response to AS605240, suggesting that the effect of this drug on Myc targets most likely occurred by posttranscriptional inhibition of Myc. Accordingly, Myc protein levels were downregulated in response to AS605240.

Glucocorticoids play a role of great importance in contemporary ALL treatment protocols. Early response to glucocorticoids is an informative prognostic factor in childhood ALL [54] and glucocorticoid resistance is a well-documented feature of relapse [17, 55]. Glucocorticoid resistance was previously associated with a proliferative phenotype involving upregulation of glycolysis, cholesterol biosynthesis, and activation of PI3K/Akt/mTOR and Myc signaling pathways [39, 56]. Indeed, fast growing T-ALL cell lines are more resistant to both prednisolone and dexamethasone [39]. Previous studies observed a synergistic interaction between rapamycin and dexamethasone in T-ALL [16, 57]. Moreover, the PI3K inhibitor LY294002 sensitized MLL-rearranged ALL cells to prednisolone *in vitro* [58]. In the present study, we showed that PI3K inhibition promotes downregulation of metabolic and biosynthesis pathways towards a gene expression profile more typical of glucocorticoid sensitive ALL cases. The PI3K inhibitor AS605240 synergized with glucocorticoids *in vitro* and the combined use of AS605240 and dexamethasone enhanced survival of mice engrafted with T-ALL. We observed no difference between *PTEN* mutated and wild-type transplanted leukemias with respect to AS605240 effect, which raises the question if T-ALL patients may benefit from PI3K inhibition irrespectively of *PTEN* mutational status. Notably, we observed that T-ALL cell lines intrinsically more resistant to prednisolone (CCRF-CEM, HPB-ALL and Molt-4) showed weaker synergism between AS605240 and prednisolone (Figure 4a–4b). This suggests that the PI3K pathway is not the only pathway underlying glucocorticoid resistance, corroborating previous findings [59]. Recent clinical and preclinical studies have shown that RAS mutations limit the effectiveness of PI3K inhibitors [60–61].

AS605240 showed an antagonistic interaction with MTX and DNR when both drugs were administered simultaneously to cell cultures. MTX kills growing tumor cells by limiting their supply of dTTP and purine nucleotides, which leads to DNA repair defects, DNA strand breaks and apoptosis. MTX-induced DNA strand break accumulation is minimal in growth-arrested tumor cells [45]. The anthracycline DNR increases the levels of topoisomerase II:DNA covalently bound complexes, generating DNA double strand breaks and inducing apoptosis. Anthracyclines appear to be particularly toxic during S-phase, when replication forks and transcription complexes are both present [62]. Our results showed that PI3K inhibition in leukemia cell lines not only induces cell cycle arrest at G_0_/G_1_, but also decreases DNA synthesis rate during cell division. This cytostatic effect is probably responsible for the tolerance exhibited by AS605240-treated cells to MTX and DNR. Our data also showed that the antagonistic interaction with MTX and DNR could be successfully circumvented by means of proper drug scheduling in more than half of the cell lines studied *in vitro* (Figure 5).

Recent evidence shows that genes most significantly correlated with ALL resistance to prednisolone are inversely correlated with MTX resistance, and that the transcriptional networks underlying resistance to one drug may actually sensitize cells to the other. Translation was suggested as one critical pathway determining the balance between glucocorticoid and MTX sensitivity in T-ALL [63]. We now provide evidence that this disparate prednisolone/MTX transcriptional profile may be under PI3K pathway control.

In summary, our gene expression analysis indicated that PI3K activity is associated with chemotherapy resistance in T-ALL. Accordingly, PI3K inhibition sensitized T-ALL cells to glucocorticoids both *in vitro* and *in vivo*. Furthermore, our data indicate that correct scheduling will be of upmost importance when combining PI3K inhibitors with drugs targeting dividing cells, which are mainstream in current protocols against T-ALL.

## METHODS

**Ethics Statement.** Investigation has been conducted in accordance with the ethical standards and according to the Declaration of Helsinki and according to national and international guidelines. The use of patient samples was approved by the FCM/UNICAMP Research Ethics Committee (CAAE: 0014.0.144.146–08) and informed consent was obtained from parents. The use of the xenograft animal model was approved by the CEEA/UNICAMP Animal Experimentation Ethics Committee (Protocol 1766–1).

**Synthesis of AS605240.** The water-soluble potassium salt of AS605240 was synthesized according to patent WO 2004007491. Identity and purity were confirmed by mass spectrometry and nuclear magnetic resonance.

**Primary T-ALL cells.** Bone marrow mononuclear cells were obtained from patients with newly diagnosed T-ALL accrued from 2000 to 2013 at Centro Infantil Boldrini, Campinas, Brazil. Treatment was performed according to the Brazilian GBTLI ALL-99 [41] and GBTLI ALL-2009 protocols. *NOTCH1*, *PTEN* and *IL7R* were sequenced as described elsewhere [12, 20, 64].

**Cell lines culture and survival assays.** Cell lines were cultured in RPMI-1640 medium, 10% fetal bovine serum (FBS), and penicillin/streptomycin at 37°C and 5% CO_2_. Medium was changed 12–24 h before all experiments. To measure IC_50_ values, cells (2–6 × 10^4^) were incubated for 96 h with increasing concentrations of AS605240, prednisolone, L-asparaginase, MTX or DNR. To measure Combination Index (CI) values between AS605240 and the other drugs, 2-fold ratios of the calculated IC_50_ values (0.25×, 0.5×, 1×, 2× or 4×) of each drug were used either in combination or as single drugs. Cells were treated for a total of 96 h. When drug scheduling was applied, one of the drugs was added 48 h after the start of the experiment. Cell viability was measured by the MTT assay. IC_50_ and CI values were calculated with the GraphPad Prism 5 (GraphPad Software, La Jolla, CA, USA) and Calcusyn (Biosoft, Cambridge, UK) [65] softwares, respectively. Calculated IC_50_ values are shown in Supplementary Table 4.

**PI3K gene expression signatures.** Cell lines (5–10 × 10^7^) were cultured with the AS605240 IC_50_ value or vehicle for 6 h, RNA was extracted by the phenol-chloroform method and purified with the RNAeasy Mini Kit (Qiagen, Inc., Valencia, CA, USA). Samples were processed with the One-Cycle Target Labeling and Control Reagents Kit (Affymetrix, Santa Clara, CA, USA) and hybridized on HG-U133 Plus 2.0 Arrays (Affymetrix). Cryopreserved primary T-ALL cells were thawed, washed in RPMI-1640 twice and viable cells were isolated with the Dead Cell Removal Kit (Miltenyi, Bergisch Gladbach, Germany). Approximately 3 × 10^6^ cells were cultured with 20 μM of AS605240 or vehicle for 6 h in AIM-V medium (Invitrogen, Carlsbad, CA, USA) supplemented with 2 mM glutamine, 10% FBS, and penicillin/streptomycin at 37°C and 5% CO_2_. RNA was extracted with the Illustra RNAspin Mini RNA Isolation Kit (GE Healthcare, Little Chalfont, UK), processed with the GeneChip WT cDNA Synthesis and Amplification Kit (Affymetrix) and hybridized on Human Gene 1.0 ST Arrays (Affymetrix). Expression values were obtained with the iterPLIER+16 algorithm and expressed in a log2 scale. Paired Limma analysis (http://www.bioconductor.org/packages/2.12/bioc/html/limma.html) was used to obtain differentially expressed genes with FDR adjusted *p*-value < 0.05 and Fold-change > 1.5. Cells of each patient or cell line treated with AS605240 or vehicle were assigned as counterparts for the paired analysis. GSEA analysis (http://www.broadinstitute.org/gsea/) [66] was performed with 1000 phenotype permutations and default settings. Only probesets/transcript clusters annotated with a Gene Symbol were used in the analysis. For Connectivity Map analysis (http://www.broadinstitute.org/cmap/) [67], only probesets identical in HG-U133A and HG-U133 Plus 2.0 arrays were used. Gene sets were considered significantly regulated when *p* < 0.05 and FDR < 0.25. Ingenuity Pathways Analysis (Ingenuity Systems, Redwood City, CA, USA) was performed with the Gene Symbols of AS605240 responsive genes. Principal Component Analysis was performed with the MultiExperiment Viewer (http://www.tm4.org/mev.html).

**Gene expression of diagnostic T-ALL.** Total RNA was extracted from T-ALL lysates preserved in guanidine isothiocyanate solution using the Illustra RNAspin Mini RNA Isolation Kit (GE Healthcare). RNA samples were processed with the WT Expression Kit (Ambion, Austin, TX, USA) and GeneChip WT Terminal Labeling and Controls Kit (Affymetrix), and hybridized on Human Gene 1.0 ST Arrays. Expression values were obtained with the iterPLIER+16 algorithm and expressed in a log2 scale. GSEA analysis was performed with 1000 gene set permutations. All array files used in this study are publicly available through the Gene Expression Omnibus (GEO) database under the accession GSE51001. For clinical and biological data, refer to Supplementary Table 3.

**Quantitative PCR.** RNA samples of T-ALL primary cells treated with AS605240 or vehicle *in vitro* were submitted to cDNA synthesis with the ImProm-II Reverse Transcription System (Promega, Madison, USA) and quantitative PCR with HOT FIREPol EvaGreen qPCR Mix Plus (Solis BioDyne, Tartu, Estonia) in a 7500 Fast Real-Time PCR System (Life Technologies, Carlsbad, USA). Expression was calculated relative to *ABL* expression. Primers used were: *ALOX5AP* (forward TAGGAGAGAGAACGCA GA, reverse AGGAACAGGAAGAGTATGA), *BCAT1* (forward AGAAGAAGAACTGGCAAC, reverse CACCTTAAATTCACCCCAC), *LDHA* (forward TTCACCCATTAAGCTGTC, reverse CAACATTCATTCCACTCC), *PRDX4* (forward GTCCAA CTGAAATTATCGCT, reverse CCAGGCCAAATGGG TAAA) and *ABL* (forward TGGAGATAACACTCTA AGCATAACTAAAGGT, reverse GATGTAGTTGCTTGG GACCCA) [68].

**Cell signaling assays.** PIP_3_ quantification of Jurkat cells (3 × 10^7^) treated for 8 h with AS605240 IC_50_ value or vehicle was performed in duplicates using the K-2500 PIP_3_ Mass ELISA Kit (Echelon Biosciences, Salt Lake City, UT, USA). Phospho-Akt quantification of Jurkat and Molt-4 cells treated for 24 h with increasing concentrations of AS605240 or vehicle was performed using the Akt [pS473] ELISA Kit (Invitrogen). Western blotting analysis of Jurkat cells treated with AS605240 IC_50_ values or vehicle was performed using antibodies against Akt (#4691, Cell Signaling Technology, Beverly, MA, USA), p-Akt (#4060, Cell Signaling Technology), Myc (N-262, Santa Cruz Biotechnology, Santa Cruz, CA, USA) and β-actin (IM-0075, Rhea Biotech, Campinas, Brazil).

**Apoptosis analysis**. Jurkat cells (1 × 10^6^) treated for 8 h with AS605240 IC_50_ value or vehicle and primary T-ALL cells (1.5 × 10^6^) treated for 6 h with 20 μM of AS605240 or vehicle were analyzed for apoptosis. Cells were either stained with PE Rabbit Anti-Active Caspase-3 (Becton Dickinson) according to manufacturer's instructions, or incubated at 37°C for 15 minutes with FITC-conjugated Annexin-V (Invitrogen Corporation) and PI 5 μg/mL in Annexin-V binding buffer. Cells were analyzed in a FACSCalibur flow cytometer.

**Cell cycle and Brdu incorporation assays**. For cell cycle analysis, Molt-4 cells (2 × 10^5^) were treated with the IC_50_ values of AS605240, MTX and/or vehicle for 24 h. Cells were fixed in 70% ethanol, washed with PBS and incubated at 37°C for 15 min in 1 mL PI Buffer (0.1% Triton X-100, 0.2 mg/mL RNAse, and 20 μg/mL PI, in PBS). Ten thousand events were collected in a FACSCalibur flow cytometer and data was analyzed with the ModFit LT 3.3 software (Verity Software House, Topsham, ME, USA). For Brdu incorporation assays, Molt-4, P12-ICHIKAWA and Jurkat cells (1 × 10^6^) were treated for 24 h with AS605240 IC_50_ values or vehicle, pulsed for 30 min with 10 μM BrdU and processed with the BrdU Flow Citometry Assay Kit (Becton Dickinson). Events (2 × 10^4^) were analyzed in a FACSCanto flow cytometer (Becton Dickinson).

***In vivo* experiments.** Primary T-ALL cells were thawed, washed with PBS and 1 × 10^7^ cells were injected in NOD/SCID (NOD.CB17-Prkdc_scid_/J) mice (The Jackson Laboratory, Bar Harbor, ME, USA) via the tail vein. After 10–12 weeks, successfully engrafted mice were sacrificed, T-ALL cells were collected from spleen and liver and 1 × 10^7^ cells were immediately injected in higher number of non-irradiated mice for the following experiments. For disease progression experiments, mice received 20 mg/Kg of AS605240 (*n* = 5) or vehicle (*n* = 12), intraperitoneally twice a day, 5 days a week, starting on the eleventh day after injection of leukemia cells. Number of human CD45+ cells in the peripheral blood after red blood cells lysis was regularly measured by flow cytometry and exponential growth curves were compared with the F test of the best-fit K values. For survival analysis experiments, mice were randomly distributed into the different treatment groups (5 animals per group) when human CD45+ reached ≥ 0.5% of peripheral blood cells in half of the animals. Mice were treated with 30 mg/Kg of AS605240 and/or 5 mg/Kg of dexamethasone, intraperitoneally once a day, 5 days a week, for 35 days. Kaplan-Meier survival curves were compared using the log-rank test with the GraphPad Prism 5 software.

## SUPPLEMENTARY FIGURES AND TABLES









## References

[1] Pui C, Relling MV, Downing JR (2004). Acute lymphoblastic leukemia. N. Engl. J. Med.

[2] Pui C, Evans WE (2006). Treatment of acute lymphoblastic leukemia. N. Engl. J. Med.

[3] Huang WC, Hung MC (2009). Induction of Akt activity by chemotherapy confers acquired resistance. J. Formos. Med. Assoc.

[4] Vivanco I, Sawyers CL (2002). The phosphatidylinositol 3-Kinase AKT pathway in human cancer. Nat. Rev. Cancer.

[5] McManus EJ, Alessi DR (2002). TSC1-TSC2: a complex tale of PKB-mediated S6K regulation. Nat. Cell Biol.

[6] Cantley LC (2002). The phosphoinositide 3-kinase pathway. Science.

[7] Neri LM, Cani A, Martelli AM, Simioni C, Junghanss C, Tabellini G, Ricci F, Tazzari PL, Pagliaro P, McCubrey JA, Capitani S (2013). Targeting the PI3K/Akt/mTOR signaling pathway in B-precursor acute lymphoblastic leukemia and its therapeutic potential. Leukemia.

[8] Schade AE, Powers JJ, Wlodarski MW, Maciejewski JP (2006). Phosphatidylinositol-3-phosphate kinase pathway activation protects leukemic large granular lymphocytes from undergoing homeostatic apoptosis. Blood.

[9] Xu Q, Simpson SE, Scialla TJ, Bagg A, Carroll M (2003). Survival of acute myeloid leukemia cells requires PI3 kinase activation. Blood.

[10] Tazzari PL, Cappellini A, Ricci F, Evangelisti C, Papa V, Grafone T, Martinelli G, Conte R, Cocco L, McCubrey JA, Martelli AM (2007). Multidrug resistance-associated protein 1 expression is under the control of the phosphoinositide 3 kinase/Akt signal transduction network in human acute myelogenous leukemia blasts. Leukemia.

[11] Silva A, Gírio A, Cebola I, Santos CI, Antunes F, Barata JT (2011). Intracellular reactive oxygen species are essential for PI3K/Akt/mTOR-dependent IL-7-mediated viability of T-cell acute lymphoblastic leukemia cells. Leukemia.

[12] Zenatti PP, Ribeiro D, Li W, Zuurbier L, Silva MC, Paganin M, Tritapoe J, Hixon JA, Silveira AB, Cardoso BA, Sarmento LM, Correia N, Toribio ML (2011). Oncogenic IL7R gain-of-function mutations in childhood T-cell acute lymphoblastic leukemia. Nat Genet.

[13] Gutierrez A, Sanda T, Grebliunaite R, Carracedo A, Salmena L, Ahn Y, Dahlberg S, Neuberg D, Moreau LA, Winter SS, Larson R, Zhang J, Protopopov A (2009). High frequency of PTEN, PI3K, and AKT abnormalities in T-cell acute lymphoblastic leukemia. Blood.

[14] Mavrakis KJ, Van Der Meulen J, Wolfe AL, Liu X, Mets E, Taghon T, Khan AA, Setty M, Setti M, Rondou P, Vandenberghe P, Delabesse E, Benoit Y (2011). A cooperative microRNA-tumor suppressor gene network in acute T-cell lymphoblastic leukemia (T-ALL). Nat Genet.

[15] Silva A, Yunes JA, Cardoso BA, Martins LR, Jotta PY, Abecasis M, Nowill AE, Leslie NR, Cardoso AA, Barata JT (2008). PTEN posttranslational inactivation and hyperactivation of the PI3K/Akt pathway sustain primary T cell leukemia viability. J Clin Invest.

[16] Zhang C, Ryu YK, Chen TZ, Hall CP, Webster DR, Kang MH (2012). Synergistic activity of rapamycin and dexamethasone *in vitro* and *in vivo* in acute lymphoblastic leukemia via cell-cycle arrest and apoptosis. Leuk Res.

[17] Kaspers GJL, Wijnands JJM, Hartmann R, Huismans L, Loonen AH, Stackelberg A, Henze G, Pieters R, Hählen K, Van Wering ER, Veerman AJP (2005). Immunophenotypic cell lineage and *in vitro* cellular drug resistance in childhood relapsed acute lymphoblastic leukaemia. Eur. J. Cancer.

[18] Gu L, Gao J, Li Q, Zhu YP, Jia CS, Fu RY, Chen Y, Liao QK, Ma Z (2008). Rapamycin reverses NPM-ALK-induced glucocorticoid resistance in lymphoid tumor cells by inhibiting mTOR signaling pathway, enhancing G1 cell cycle arrest and apoptosis. Leukemia.

[19] Gu L, Zhou C, Liu H, Gao J, Li Q, Mu D, Ma Z (2010). Rapamycin sensitizes T-ALL cells to dexamethasone-induced apoptosis. J. Exp. Clin. Cancer Res.

[20] Jotta PY, Ganazza MA, Silva A, Viana MB, da Silva MJ, Zambaldi LJG, Barata JT, Brandalise SR, Yunes JA (2010). Negative prognostic impact of PTEN mutation in pediatric T-cell acute lymphoblastic leukemia. Leukemia.

[21] Zuurbier L, Petricoin EF, Vuerhard MJ, Calvert V, Kooi C, Buijs-Gladdines JGC AM, Smits WK, Sonneveld E, Veerman AJP, Kamps WA, Horstmann M, Pieters R, Meijerink JPP (2012). The significance of PTEN and AKT aberrations in pediatric T-cell acute lymphoblastic leukemia. Haematologica.

[22] Bandapalli OR, Zimmermann M, Kox C, Stanulla M, Schrappe M, Ludwig WD, Koehler R, Muckenthaler MU, Kulozik AE (2013). NOTCH1 activation clinically antagonizes the unfavorable effect of PTEN inactivation in BFM-treated children with precursor T-cell acute lymphoblastic leukemia. Haematologica.

[23] Camps M, Rückle T, Ji H, Ardissone V, Rintelen F, Shaw J, Ferrandi C, Chabert C, Gillieron C, Françon B, Martin T, Gretener D, Perrin D (2005). Blockade of PI3Kgamma suppresses joint inflammation and damage in mouse models of rheumatoid arthritis. Nat Med.

[24] Barber DF, Bartolomé A, Hernandez C, Flores JM, Redondo C, Fernandez-Arias C, Camps M, Rückle T, Schwarz MK, Rodríguez S, Martinez AC, Balomenos D, Rommel C (2005). PI3Kgamma inhibition blocks glomerulonephritis and extends lifespan in a mouse model of systemic lupus. Nat. Med.

[25] De Keersmaecker K, Atak ZK, Li N, Vicente C, Patchett S, Girardi T, Gianfelici V, Geerdens E, Clappier E, Porcu M, Lahortiga I, Lucà R, Yan J (2013). Exome sequencing identifies mutation in CNOT3 and ribosomal genes RPL5 and RPL10 in T-cell acute lymphoblastic leukemia. Nat Genet.

[26] Scupoli MT, Perbellini O, Krampera M, Vinante F, Cioffi F, Pizzolo G (2007). Interleukin 7 requirement for survival of T-cell acute lymphoblastic leukemia and human thymocytes on bone marrow stroma. Haematologica.

[27] Rots MG, Pieters R, Kaspers GJ, van Zantwijk CH, Noordhuis P, Mauritz R, Veerman AJ, Jansen G, Peters GJ (1999). Differential methotrexate resistance in childhood T-versus common/preB-acute lymphoblastic leukemia can be measured by an in situ thymidylate synthase inhibition assay, but not by the MTT assay. Blood.

[28] Menssen A, Hermeking H (2002). Characterization of the c-MYC-regulated transcriptome by SAGE: identification and analysis of c-MYC target genes. Proc Natl Acad Sci U.S.A.

[29] Zeller KI, Jegga AG, Aronow BJ, O'Donnell KA, Dang CV (2003). An integrated database of genes responsive to the Myc oncogenic transcription factor: identification of direct genomic targets. Genome Biol.

[30] Schuhmacher M, Kohlhuber F, Hölzel M, Kaiser C, Burtscher H, Jarsch M, Bornkamm GW, Laux G, Polack A, Weidle UH, Eick D (2001). The transcriptional program of a human B cell line in response to Myc. Nucleic Acids Res.

[31] Bild AH, Yao G, Chang JT, Wang Q, Potti A, Chasse D, Joshi MB, Harpole D, Lancaster JM, Berchuck A, Olson JA, Marks JR, Dressman HK (2006). Oncogenic pathway signatures in human cancers as a guide to targeted therapies. Nature.

[32] Lee LA, Dang CV (2006). Myc target transcriptomes. Curr Top Microbiol Immunol.

[33] King B, Trimarchi T, Reavie L, Xu L, Mullenders J, Ntziachristos P, Aranda-Orgilles B, Perez-Garcia A, Shi J, Vakoc C, Sandy P, Shen SS, Ferrando A (2013). The ubiquitin ligase FBXW7 modulates leukemia-initiating cell activity by regulating MYC stability. Cell.

[34] Wang H, Zou J, Zhao B, Johannsen E, Ashworth T, Wong H, Pear WS, Schug J, Blacklow SC, Arnett KL, Bernstein BE, Kieff E, Aster JC (2011). Genome-wide analysis reveals conserved and divergent features of Notch1/RBPJ binding in human and murine T-lymphoblastic leukemia cells. Proc. Natl. Acad. Sci. U.S.A.

[35] Sharma VM, Calvo JA, Draheim KM, Cunningham LA, Hermance N, Beverly L, Krishnamoorthy V, Bhasin M, Capobianco AJ, Kelliher MA (2006). Notch1 contributes to mouse T-cell leukemia by directly inducing the expression of c-myc. Mol Cell Biol.

[36] Zhou W, Feng X, Ren C, Jiang X, Liu W, Huang W, Liu Z, Li Z, Zeng L, Wang L, Zhu B, Shi J, Liu J (2013). Over-expression of BCAT1, a c-Myc target gene, induces cell proliferation, migration and invasion in nasopharyngeal carcinoma. Mol Cancer.

[37] Schramm A, Schulte JH, Klein-Hitpass L, Havers W, Sieverts H, Berwanger B, Christiansen H, Warnat P, Brors B, Eils J, Eils R, Eggert A (2005). Prediction of clinical outcome and biological characterization of neuroblastoma by expression profiling. Oncogene.

[38] Oberthuer A, Berthold F, Warnat P, Hero B, Kahlert Y, Spitz R, Ernestus K, König R, Haas S, Eils R, Schwab M, Brors B, Westermann F (2006). Customized oligonucleotide microarray gene expression-based classification of neuroblastoma patients outperforms current clinical risk stratification. J. Clin. Oncol.

[39] Beesley AH, Firth MJ, Ford J, Weller RE, Freitas JR, Perera KU, Kees UR (2009). Glucocorticoid resistance in T-lineage acute lymphoblastic leukaemia is associated with a proliferative metabolism. Br. J. Cancer.

[40] Madden EA, Bishop EJ, Fiskin AM, Melnykovych G (1986). Possible role of cholesterol in the susceptibility of a human acute lymphoblastic leukemia cell line to dexamethasone. Cancer Res.

[41] Brandalise SR, Pinheiro VR, Aguiar SS, Matsuda EI, Otubo R, Yunes JA, Pereira WV, Carvalho EG, Cristofani LM, Souza MS, Lee ML, Dobbin JA, Pombo-de-Oliveira MS (2010). Benefits of the intermittent use of 6-mercaptopurine and methotrexate in maintenance treatment for low-risk acute lymphoblastic leukemia in children: randomized trial from the Brazilian Childhood Cooperative Group—protocol ALL-99. J. Clin. Oncol.

[42] Ashraf J, Thompson E (1993). Identification of the activation-labile gene: a single point mutation in the human glucocorticoid receptor presents as two distinct receptor phenotypes. Mol. Endocrinol.

[43] Ruiz M, Lind U, Gåfvels M (2001). Characterization of two novel mutations in the glucocorticoid receptor gene in patients with primary cortisol resistance. Clin. Endocrinol.

[44] Rajagopalan PTR, Zhang Z, McCourt L, Dwyer M, Benkovic SJ, Hammes GG (2002). Interaction of dihydrofolate reductase with methotrexate: ensemble and single-molecule kinetics. Proc. Natl. Acad. Sci. U.S.A.

[45] Li JC, Kaminskas E (1984). Accumulation of DNA strand breaks and methotrexate cytotoxicity. Proc. Natl. Acad. Sci. U.S.A.

[46] Nicholson KM, Anderson NG (2002). The protein kinase B/Akt signalling pathway in human malignancy. Cell Signal.

[47] Yamamoto S, Tomita Y, Hoshida Y, Morooka T, Nagano H, Dono K, Umeshita K, Sakon M, Ishikawa O, Ohigashi H, Nakamori S, Monden M, Aozasa K (2004). Prognostic significance of activated Akt expression in pancreatic ductal adenocarcinoma. Clin Cancer Res.

[48] Ermoian RP, Furniss CS, Lamborn KR, Basila D, Berger MS, Gottschalk AR, Nicholas MK, Stokoe D, Haas-Kogan DA (2002). Dysregulation of PTEN and protein kinase B is associated with glioma histology and patient survival. Clin Cancer Res.

[49] Fruman DA, Cantley LC (2002). Phosphoinositide 3-kinase in immunological systems. Semin Immunol.

[50] Subramaniam PS, Whye DW, Efimenko E, Chen J, Tosello V, De Keersmaecker K, Kashishian A, Thompson MA, Castillo M, Cordon-Cardo C, Davé UP, Ferrando A, Lannutti BJ (2012). Targeting nonclassical oncogenes for therapy in T-ALL. Cancer Cell.

[51] Bonnet M, Loosveld M, Montpellier B (2011). Posttranscriptional deregulation of MYC via PTEN constitutes a major alternative pathway of MYC activation in T-cell acute lymphoblastic leukemia. Blood.

[52] Csibi A, Lee G, Yoon S-O, Tong H, Ilter D, Elia I, Fendt S-M, Roberts TM, Blenis J (2014). The mTORC1/S6K1 pathway regulates glutamine metabolism through the eIF4B-dependent control of c-Myc translation. Curr Biol.

[53] Weng AP, Millholland JM, Yashiro-Ohtani Y, Arcangeli ML, Lau A, Wai C, Del Bianco C, Rodriguez CG, Sai H, Tobias J, Li Y, Wolfe MS, Shachaf C (2006). c-Myc is an important direct target of Notch1 in T-cell acute lymphoblastic leukemia/lymphoma. Genes Dev.

[54] Dördelmann M, Reiter A, Borkhardt A, Ludwig WD, Götz N, Viehmann S, Gadner H, Riehm H, Schrappe M (1999). Prednisone Response Is the Strongest Predictor of Treatment Outcome in Infant Acute Lymphoblastic Leukemia. Blood.

[55] Klumper E, Pieters R, Veerman AJ, Huismans DR, Loonen AH, Hählen K, Kaspers GJ, van Wering ER, Hartmann R, Henze G (1995). *In vitro* cellular drug resistance in children with relapsed/refractory acute lymphoblastic leukemia. Blood.

[56] Hulleman E, Kazemier KM, Holleman A, VanderWeele DJ, Rudin CM, Broekhuis MJC, Evans WE, Pieters R, Den Boer ML (2009). Inhibition of glycolysis modulates prednisolone resistance in acute lymphoblastic leukemia cells. Blood.

[57] Batista A, Barata JT, Raderschall E, Sallan SE, Carlesso N, Nadler LM, Cardoso AA (2011). Targeting of active mTOR inhibits primary leukemia T cells and synergizes with cytotoxic drugs and signaling inhibitors. Exp Hematol.

[58] Spijkers-Hagelstein JAP, Pinhanços SS, Schneider P, Pieters R, Stam RW (2013). Chemical genomic screening identifies LY294002 as a modulator of glucocorticoid resistance in MLL-rearranged infant ALL. Leukemia.

[59] Wei G, Twomey D, Lamb J, Schlis K, Agarwal J, Stam RW, Opferman JT, Sallan SE, den Boer ML, Pieters R, Golub TR, Armstrong SA (2006). Gene expression-based chemical genomics identifies rapamycin as a modulator of MCL1 and glucocorticoid resistance. Cancer Cell.

[60] Engelman J, Chen L, Tan X (2008). Effective use of PI3K and MEK inhibitors to treat mutant Kras G12D and PIK3CA H1047R murine lung cancers. Nat Med.

[61] Janku F, Tsimberidou AM, Garrido-Laguna I, Wang X, Luthra R, Hong DS, Naing A, Falchook GS, Moroney JW, Piha-Paul SA, Wheler JJ, Moulder SL, Fu S (2011). PIK3CA mutations in patients with advanced cancers treated with PI3K/AKT/mTOR axis inhibitors. Mol Cancer Ther.

[62] Kaufmann SH (1998). Cell death induced by topoisomerase-targeted drugs: more questions than answers. Biochim Biophys Acta.

[63] Beesley AH, Firth MJ, Anderson D, Samuels AL, Ford J, Kees UR (2013). Drug-gene modeling in pediatric T-cell acute lymphoblastic leukemia highlights importance of 6-mercaptopurine for outcome. Cancer Res.

[64] Silva A, Jotta PY, Silveira AB, Ribeiro D, Brandalise SR, Yunes JA, Barata JT (2010). Regulation of PTEN by CK2 and Notch1 in primary T-cell acute lymphoblastic leukemia: rationale for combined use of CK2- and gamma-secretase inhibitors. Haematologica.

[65] Chou T (2006). Theoretical basis, experimental design, and computerized simulation of synergism and antagonism in drug combination studies. Pharmacol Rev.

[66] Subramanian A, Tamayo P, Mootha VK, Mukherjee S, Ebert BL, Gillette MA, Paulovich A, Pomeroy SL, Golub TR, Lander ES, Mesirov JP (2005). Gene set enrichment analysis: a knowledge-based approach for interpreting genome-wide expression profiles. Proc. Natl. Acad. Sci. U.S.A.

[67] Lamb J (2007). The Connectivity Map: a new tool for biomedical research. Nat. Rev. Cancer.

[68] Beillard E, Pallisgaard N, van der Velden VHJ, Bi W, Dee R, van der Schoot E, Delabesse E, Macintyre E, Gottardi E, Saglio G, Watzinger F, Lion T, van Dongen JJM (2003). Evaluation of candidate control genes for diagnosis and residual disease detection in leukemic patients using “real-time” quantitative reverse-transcriptase polymerase chain reaction (RQ-PCR) - a Europe against cancer program. Leukemia.

