# Supplementary material for: PI3K inhibition synergizes with glucocorticoids but antagonizes with methotrexate in T-cell acute lymphoblastic leukemia
Source: Oncotarget. 2015 Apr 1;6(15):13105–18. doi: 10.18632/oncotarget.3524 (PMC4537002; doi:10.18632/oncotarget.3524)
Supplement: Supplementary file 1 [file oncotarget-06-13105-s001.pdf]

SUPPLEMENTARY FIGURES AND TABLES

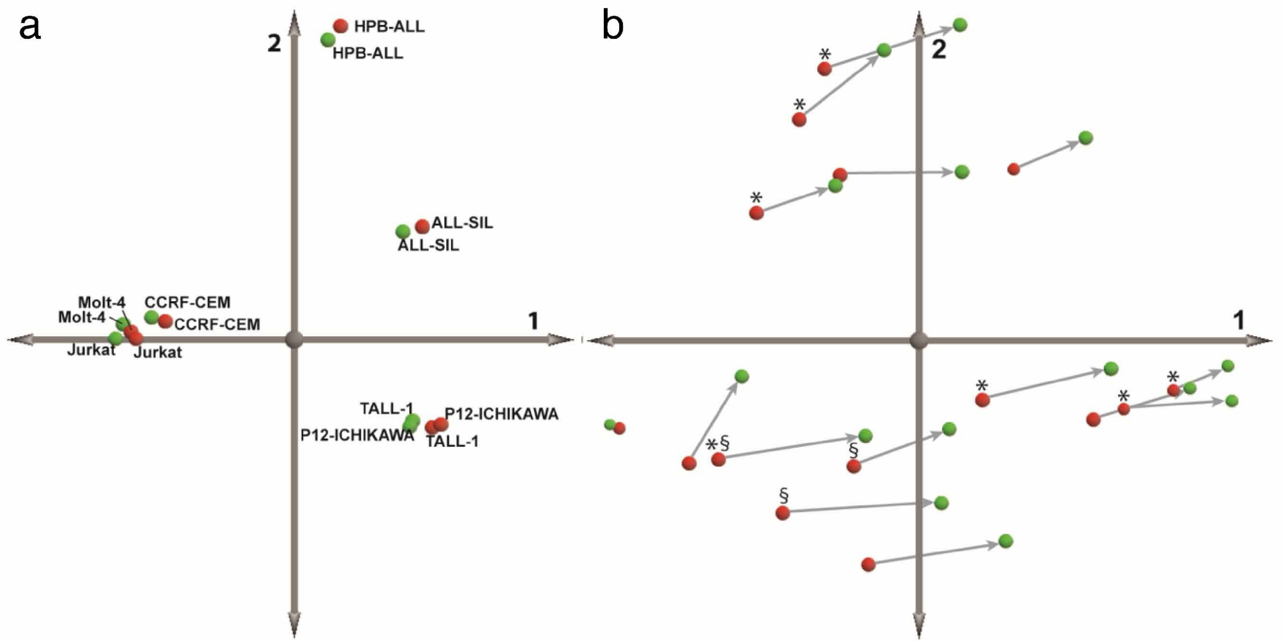

Supplementary Figure S1: T-ALL samples responded similarly to PI3K inhibition, irrespectively of *PTEN* and *NOTCH1* mutational status. Principal component analysis of global gene expression data of T-ALL cell lines **(a)** and primary cells **(b)** treated for 6 h with AS605240 (in red) or vehicle (in green). Most samples responded similarly to AS605240 treatment, evidenced by a shift in the same direction of treated samples in comparison to controls (arrows). *NOTCH1* (*) and *PTEN* mutated patients (§) are indicated for control samples only. Gene expression profile was obtained with HG-U133 Plus 2.0 arrays for T-ALL cell lines and Human Gene 1.0 ST Arrays for T-ALL primary cells.

Drug	Score	p-value	CMap plot
wortmannin	0.807	0.000	
LY-294002	0.766	0.000	
sirolimus	0.843	0.000	

Supplementary Figure S2: AS605240-derived gene expression signature correlated with signatures of other PI3K pathway inhibitors. Connectivity Map analysis using AS605240 responsive genes in T-ALL cell lines. AS605240 modulated gene expression similarly to two classical PI3K inhibitors (wortmannin and LY-294002) and the mTOR inhibitor rapamycin (sirolimus).

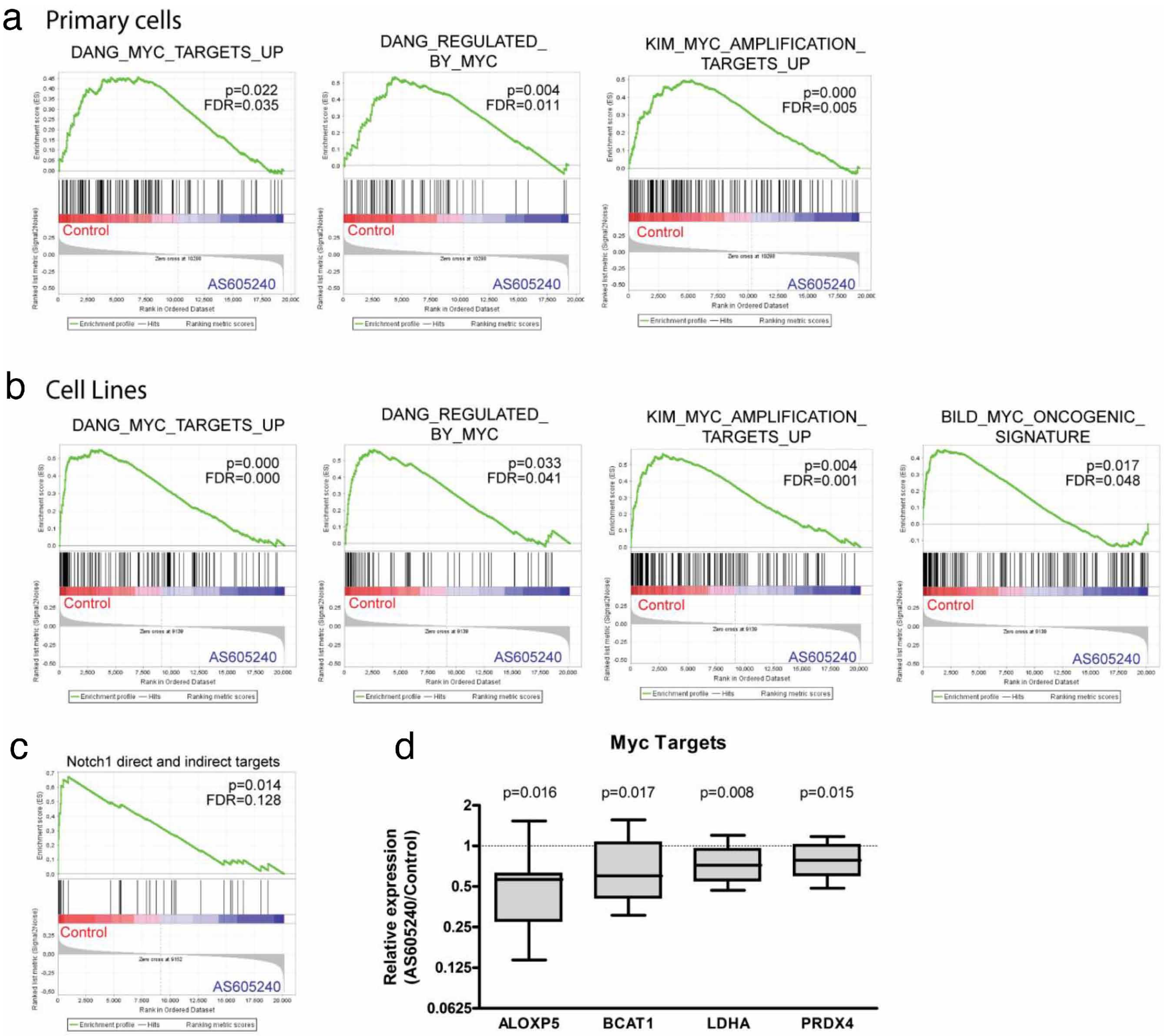

Supplementary Figure S3: AS605240 downregulated Myc targets but not Notch1 direct targets in T-ALL cells. (a–b) GSEA analysis indicating that Myc targets (Menssen et al., 2002; Zeller et al., 2003) are downregulated in T-ALL primary cells and cell lines treated with AS605240 *in vitro*. A gene set representing a Myc oncogenic signature was also downregulated by AS605240 in cell lines (Bild et al., 2006). **(c)** GSEA analysis indicating that Notch1-responsive genes (Sharma et al., 2006) are downregulated in T-ALL cell lines treated with AS605240. **(d)** Quantitative PCR showing downregulation of Myc targets *ALOX5AP* (selected from gene set KIM_MYC_AMPLIFICATION_TARGETS_UP), *BCAT1* (DANG_MYC_TARGETS_UP), *PRDX4* and *LDHA* (SCHUHMACHER_MYC_TARGETS_UP) in T-ALL primary cells in response to PI3K inhibition. Expression was calculated relative to *ABL* expression. *P*-values obtained with paired *T*-tests.

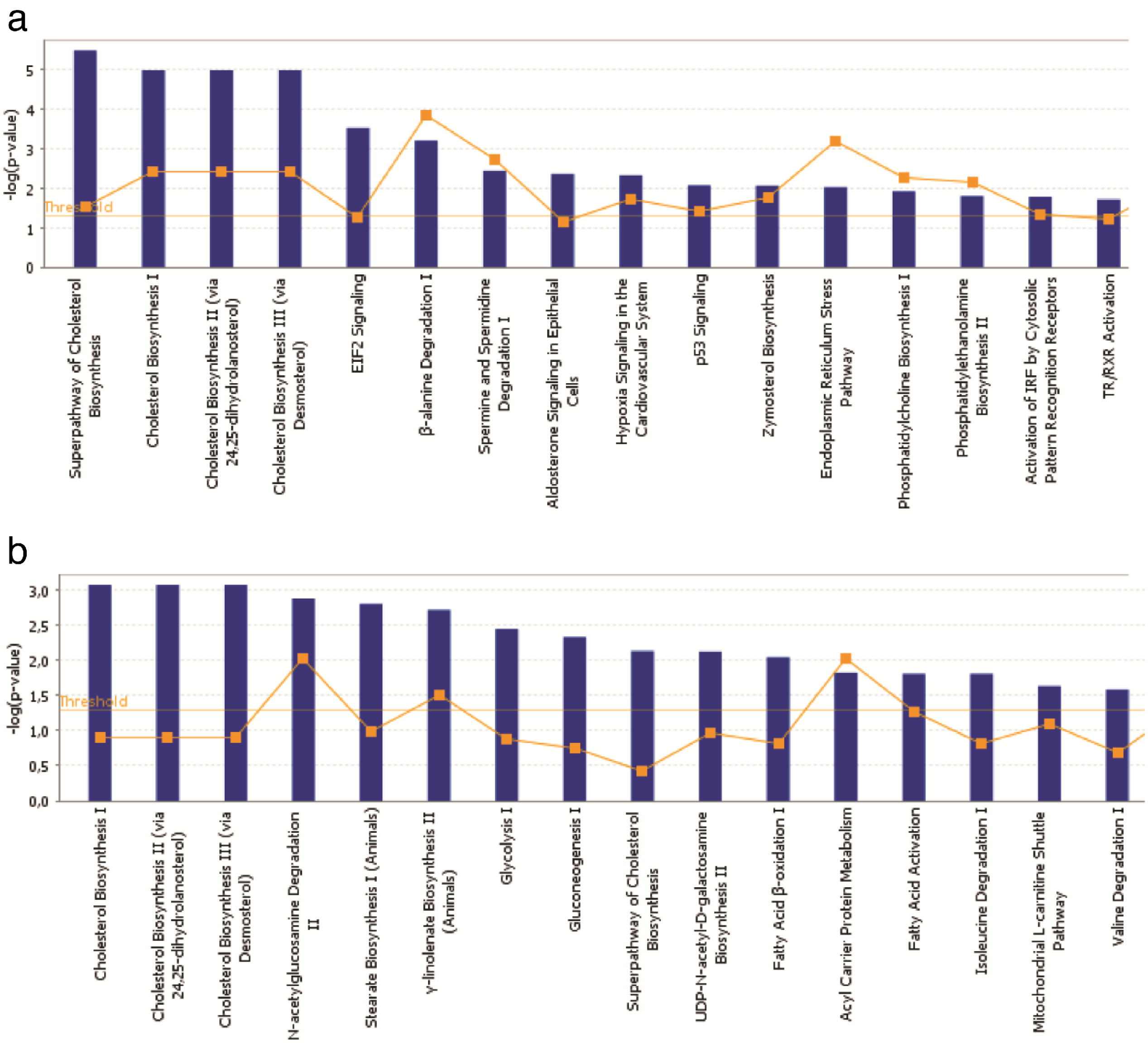

Supplementary Figure S4: AS605240 modulates the expression of genes related to cholesterol biosynthesis. Ingenuity Pathway Analysis was performed with AS605240 responsive gene in **(a)** T-ALL cell lines and **(b)** primary T-ALL cells. Top biofunctions downregulated by AS605240 in both cell lines and primary cells represented cholesterol biosynthesis pathways.

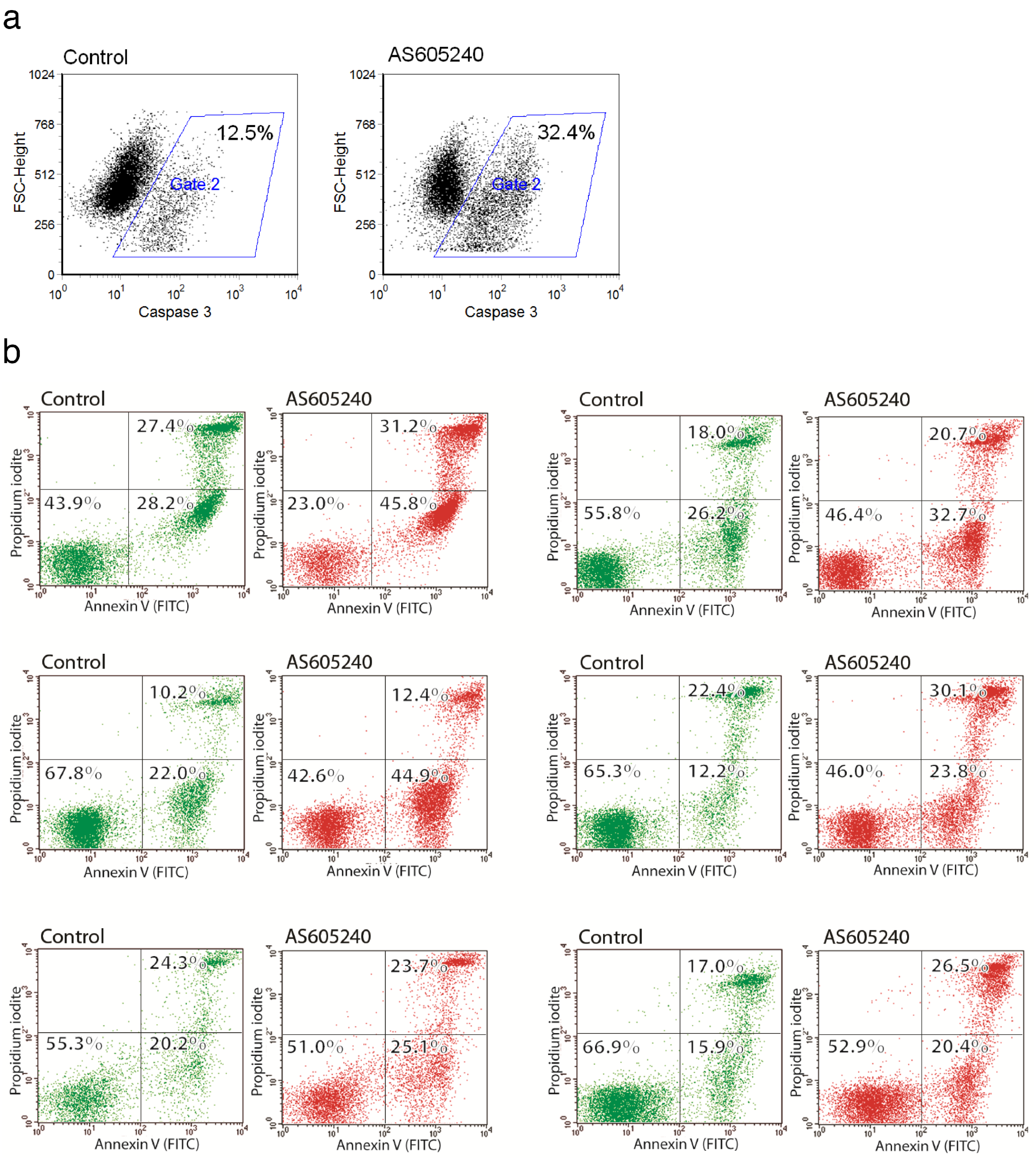

Supplementary Figure S5: AS605240 induced apoptosis in T-ALL cells *in vitro*. (a) Jurkat cells treated with AS605240 IC_{50} value for 8 h were marked with PE Rabbit Anti-Active Caspase-3 antibody and analyzed by flow cytometry. **(b)** Primary T-ALL cells treated with AS605240 or vehicle for 6 h were incubated with FITC-conjugated Annexin-V and propidium iodide and analyzed by flow cytometry. Apoptotic cells are represented in the lower right quadrants of the plots. Each pair of control and treatment plots represents data obtained with primary cells from a different T-ALL patient.

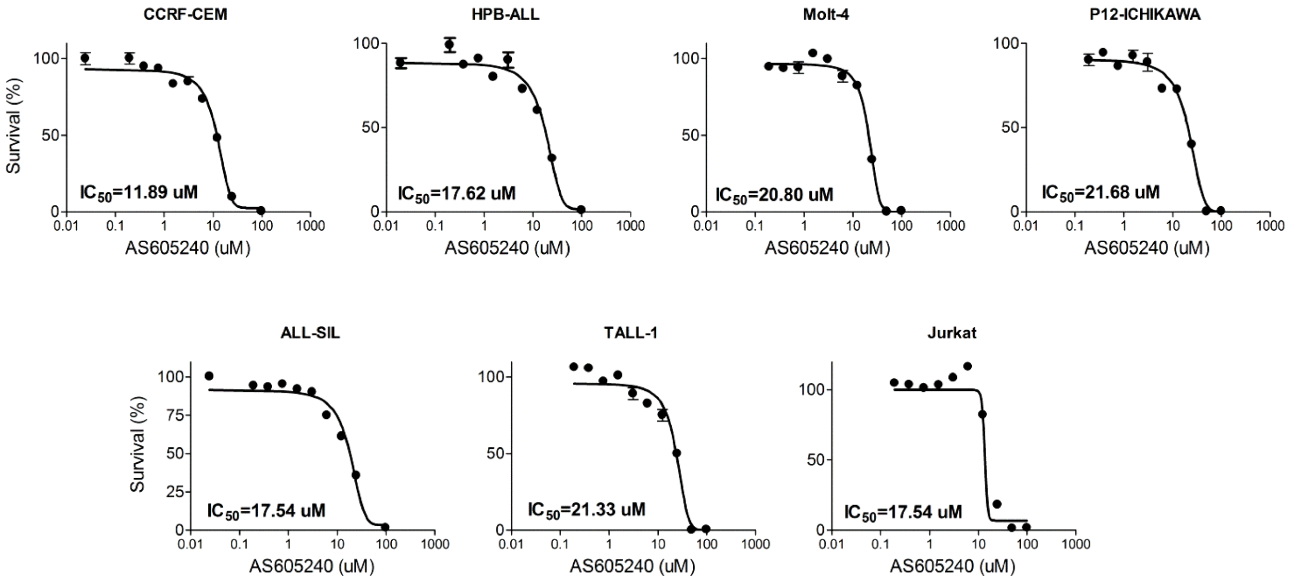

Supplementary Figure S6: IC_{50} values of AS605240 for different T-ALL cell lines. Survival of T-ALL cell lines incubated with increasing concentrations of AS605240 for 96 h, as assessed by the MTT assay. IC_{50} values were calculated with the GraphPad Prism 5 software.

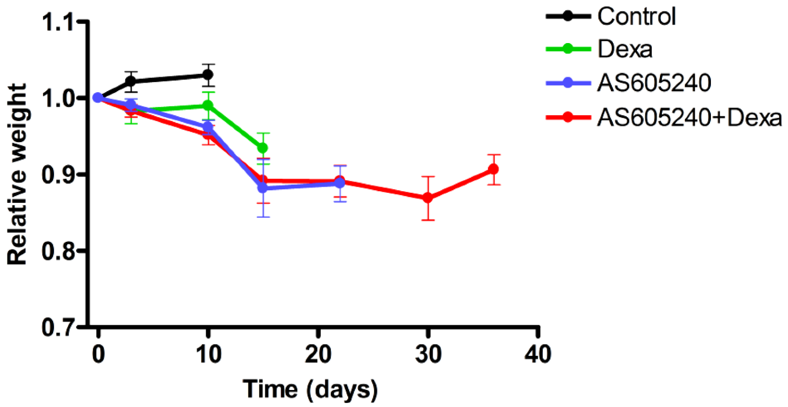

Supplementary Figure S7: Relative weight of T-ALL xenografted NOD/SCID mice treated with vehicle, AS605240 and/or dexamethasone. Relative weight was here defined as the animal's weight divided by its weight immediately before start of treatment. NOD/SCID mice were engrafted with 1x107 primary T-ALL cells via tail vein injection. Treatment started when human CD45+ accounted for more than 0.5% of peripheral blood cells after red blood cells lysis, as measured by flow cytometry. Mice were treated with 30 mg/Kg of AS605240 and/or 5 mg/Kg of dexamethasone, intraperitoneally once a day, 5 days a week.

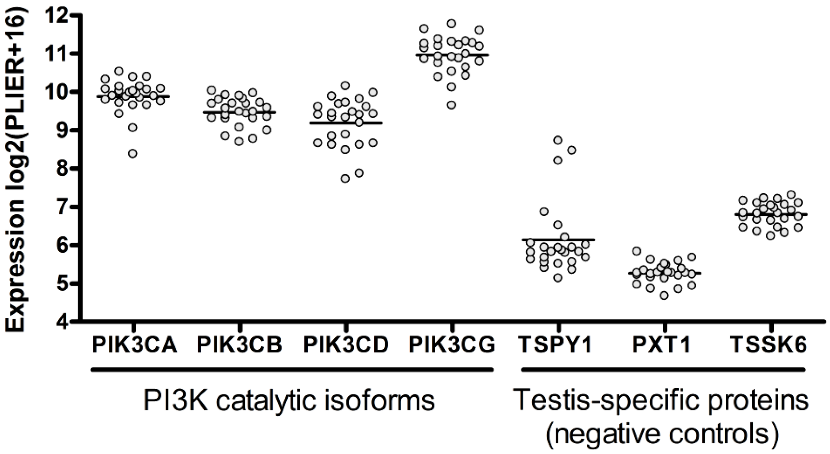

Supplementary Figure S8: All p110 catalytic subunits of PI3K (*PIK3CA*, *PIK3CB*, *PIK3CD* and *PIK3CG*) are highly expressed in primary T-ALL. Global gene expression of 24 T-ALL samples collected at diagnosis was measured with the Human Gene 1.0 ST arrays. Testis-specific protein genes are shown as negative controls (*TSPY1*, *PXT1* and *TSSK6*).

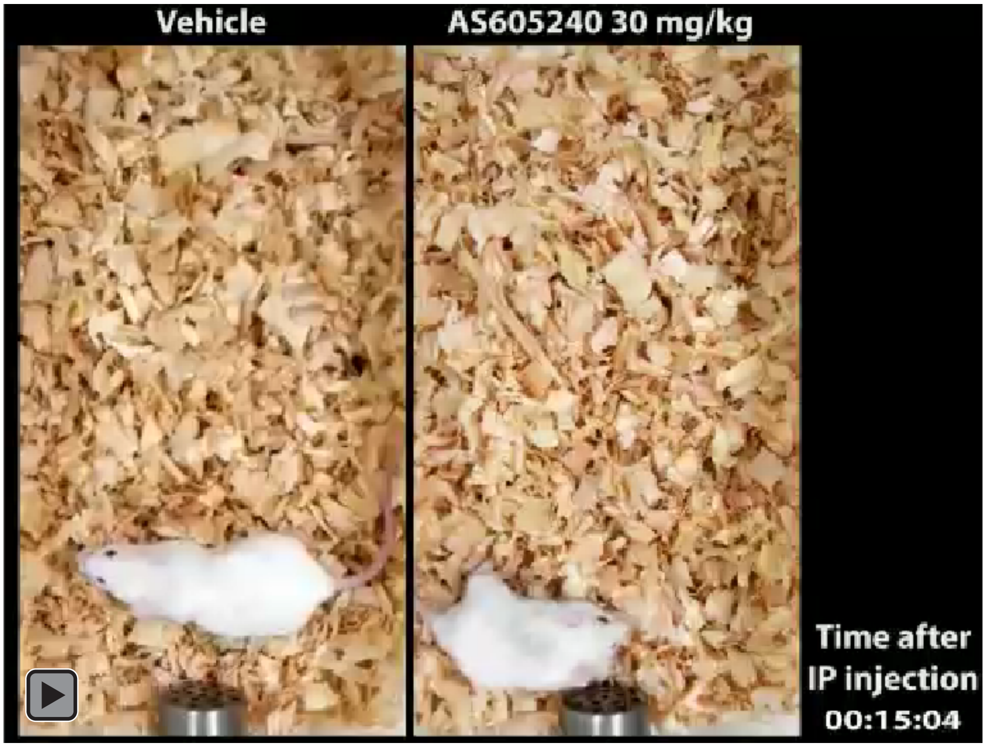

Supplementary Video:

Supplementary Table 1: Genes differentially expressed in T-ALL primary cells in response to AS605240 treatment (Limma analysis; adjusted p-value < 0.05, FC > 1.5).

Supplementary Table 2: Genes differentially expressed in T-ALL cell lines in response to AS605240 treatment (Limma analysis; adjusted p-value < 0.05, FC > 1.5).

Supplementary Table 3: Clinical and biological features of the 43 T-ALL cases included in expression microarray analysis. *NOTCH1, PTEN* and *IL7R* mutated (Mut) and wild-type (wt) samples are indicated. Immunophenotypic markers are presented as percentage of positive cells by flow cytometry.

Supplementary Table S4: values of AS605240 and chemotherapy drugs in T-ALL cell lines. Cell viability was measured by the MTT assay and IC_{50} values were calculated with the GraphPad Prism 5 software.

	IC_{50}				
	**AS605240**	**Pred**	**Asp**	**MTX**	**DNR**
**Cell line**	**(µM)**	**(ug/mL)**	**(IU/mL)**	**(nM)**	**(ug/mL)**
**CCRF-CEM**	11,89	209,5	0,123	16,11	0,233
**HPB-ALL**	17,62	111,1	0,000075	41,98	0,265
**Jurkat**	17,54	357,6	0,133	35,56	0,315
**Molt-4**	20,80	190,0	0,090	23,01	0,507
**P12-ICHIKAWA**	21,68	46,32	0,133	8,47	0,083
**ALL-SIL**	17,54	21,20	0,000012	18,32	0,112
**TALL-1**	21,33	14,39	0,111	15,84	0,111
